# Impact of the COVID-19 pandemic on number of deaths in Ghana: an application of ARIMA with intervention analysis

**DOI:** 10.3389/fpubh.2025.1649498

**Published:** 2025-09-02

**Authors:** Christian Akrong Hesse, Emmanuel Dodzi Kpeglo, Dominic Buer Boyetey, Saralees Nadarajah

**Affiliations:** ^1^Department of Economics and Actuarial Science, University of Professional Studies, Accra, Ghana; ^2^Department of Built Environment, University of Environment and Sustainable Development, Somanya, Ghana; ^3^Department of Mathematics, University of Manchester, Manchester, United Kingdom

**Keywords:** COVID-19, pandemic, time series, intervention analysis, death count

## Abstract

Over the past three years, the novel coronavirus (COVID-19) outbreak has caused millions of unexpected deaths worldwide. Ghana, like many other countries, recorded numerous COVID-19 deaths. The number of deaths from the commencement of the crisis formed a time series. This study examines the effect of the COVID-19 pandemic on the number of deaths in Ghana using intervention analysis on time series data. Initially, we generated an ARIMA (3, 1, 0) model to examine monthly death data in Ghana from January 2018 to December 2022, identifying March 2020 as the critical intervention point. March 2020 marked the commencement of the COVID-19 pandemic in Ghana. Secondly, we employed the ARIMA model to predict the post-pandemic death trend, and ultimately, we found no substantial discrepancy between the estimated and observed death count. Consequently, the analysis concluded that, despite a surge in global deaths, the estimated model indicated that the pandemic’s emergence did not result in a significant change in the number of deaths in Ghana beyond the anticipated figures based on pre-pandemic patterns.

## 1 Introduction

There is no doubt that every crisis poses a threat to life, regardless of its severity and the population affected. This threat was evident during the outbreak of the novel coronavirus (COVID-19) that caused millions of unexpected deaths (1). The death count and aggregates across countries generated a time series for the global community’s consideration. While it is assumed that a person’s health is relatively unimpaired until it fails, (2) projected that COVID-19 patients have a higher probability of death in the first two weeks, which then decreases after two weeks, then declines after two weeks. A pattern that adds to the unpredictability of the disease. Then declines after two weeks. A pattern that adds to the unpredictability of the disease. The drop is not only experienced in the number of deaths but also in the monthly peaks of COVID-19 new cases. These dynamics underscore the profound impact of the pandemic, both in terms of its unprecedented mortality burden and the valuable time-series data it produced for statistical analysis. Against this background, the present study investigates the effect of the COVID-19 pandemic on mortality levels in Ghana, employing time-series intervention analysis to assess changes over time.

The COVID-19 pandemic has caused profound and sustained impacts on global mortality, health systems, and socio-economic stability. For instance, healthcare utilization patterns for non-COVID-19 care were considerably impacted by a confluence of patient- and institution-driven factors. Patient fear of contracting the virus led many individuals to delay or avoid seeking medical care, which, as (3) highlighted, could have mental and physical health consequences. Healthcare institutions restructured their services, as noted by (4, 5), by cancelling elective procedures and appointments, and imposing restrictions that created logistical barriers for patients. A measure that created a disproportionate impact on different care types and populations (6). For example, the management of chronic diseases was significantly disrupted in Korea, while (7) observed an adverse effect on people with disabilities. A World Health Organization (WHO) survey referenced by (8) further revealed that services for non-communicable diseases were partially or completely disrupted in many countries, with a higher percentage of disruptions occurring in low- and middle-income nations. Collectively, these factors contributed to a higher risk of complications and death, as (9) pointed out, demonstrating how the pandemic’s influence on healthcare extended far beyond COVID-19 cases.

Accurate mortality forecasting is crucial for anticipating healthcare demands, guiding policy responses, and evaluating the indirect effects of the pandemic on population health. Globally, statistical and computational models, including autoregressive integrated moving average (ARIMA) methods, have been widely applied to forecast mortality and morbidity trends, offering valuable insights for short- to medium-term public health planning (10–12).

In Africa, while COVID-19’s direct mortality burden has been significant in several African countries, indirect effects such as healthcare disruption, reduced care-seeking, and socio-economic stressors have also influenced overall mortality patterns. Forecasting in such contexts requires models that can work reliably with the available data while capturing both pandemic-related and background mortality trends (13). employed the generalized extreme value distribution to model the monthly maximum of daily new cases from Benin, Burkina Faso, Cabo Verde, Côte d’Ivoire, Gambia, Ghana, Guinea, Guinea-Bissau, Liberia, Mali, Mauritania, Niger, Nigeria, Senegal, Sierra Leone, and Togo. The authors identified significant declines in the monthly maximums of daily new cases in ten of the sixteen countries: Ghana, Benin, Cabo Verde, Côte d’Ivoire, Guinea, Mauritania, Nigeria, Sierra Leone, Mali, and Senegal (14), showed that ARIMA could reliably forecast daily confirmed and death cases of COVID-19 in Nigeria, highlighting its applicability even in settings with limited resources. Here, “a COVID-19 death is defined for surveillance purposes as a death resulting from a clinically compatible illness in a probable or confirmed COVID-19 case, unless there is a clear alternative cause of death that cannot be related to COVID-19 disease” (15).

Indeed, failing health is inevitable as it remains constant over some recovery period; otherwise, death ensues (16). Research has proposed various scenarios that simulate the likelihood of deaths at any particular moment based on historical data. The Weibull distribution, according to (17), provides an optimal failure rate during surgical procedures, but the rate reduces as the person responds to treatment and the condition improves over time. The exponential distribution proffers a declining failure rate for a healthy individual over a designated period (18, 19), propose that the exponential distribution and the Weibull distribution may be inapplicable to rare cases, such as the failure rate of COVID-19 patients. Their proposal was based on the virus’s irregular failure rate and, therefore, may require a model with a non-monotonic failure rate. Following Farooq et al.’s assertion, several studies have commenced modelling the uncertainty surrounding the failure rate of the COVID-19 infection (20), predicted intertemporal mortality for COVID-19 patients utilising an exponential-like approach (21), employed a novel flexible extended Weibull model to estimate the overall number of COVID-19 fatalities, while Farooq et al. in 2022 applied the new flexible Weibull model.

In contrast to the distributional approach in the studies of COVID-19 deaths, ARIMA models have proven effective in forecasting COVID-19 deaths, as demonstrated by several studies. Researchers such as (22, 23) have demonstrated the accuracy of autoregressive integrated moving averages (ARIMA) in predicting daily and weekly case and death counts, respectively. Studies by (24, 25) highlight the use of time series metrics like mean absolute percentage error (MAPE), mean absolute error (MAE), mean absolute deviation (MAD), mean squared error (MSE), root mean squared error (RMSE), Akaike information criterion (AIC), Bayesian information criterion (BIC), autocorrelation function (ACF) and population attributable fraction (PAF) graphs to validate ARIMA’s suitability, often outperforming alternative methods like machine learning, neural network model, multiple regression, susceptible-exposed-infectious-recovered (SEIR) models (26), further substantiated ARIMA’s reliability in predicting cumulative COVID-19 deaths across heavily impacted nations, consistently surpassing benchmark forecasting techniques and indicating a general upward trend in fatalities. These findings underscore the value of ARIMA in providing accurate short-term forecasts for COVID-19 death trajectories.

Available statistics through research reveal remarkable figures regarding fatalities due to COVID-19 infection. The WHO reported a total of 6,987,831 COVID-19 deaths worldwide as of November 19, 2023. Among the total, the Americas accounted for the most considerable percentage of deaths, at 43%, followed by Europe at 32%. Africa recorded the lowest percentage of COVID-19 deaths at 3%. A study conducted by the (27) indicated that COVID-19 death rates for individuals aged 35 to 64 and those aged 65 and beyond are 1.3 times and 2.5 times greater, respectively, in the unvaccinated cohort compared to those who have received at least one booster dose (28), also discovered that deaths associated with COVID-19 were prevalent in Zambia, and the percentage of such deaths escalated with advancing age (29). South Africa documented around 20,000 fatalities and a case fatality rate of 2.7%, as reported by (30, 31). As of July 2, 2023, Ghana recorded 1,462 COVID-19 deaths (1). The puzzle in these statistics is the low number of COVID-19 death cases recorded in African nations, which have invariably larger population densities.

Literature identifies three factors that contribute to the low COVID-19 deaths in Africa. Mwananyanda et al. suggest that the low deaths may derive from inadequate data on COVID-19 deaths, which is attributed to exorbitant expenses associated with disease surveillance systems. In addition, the perception is that the younger population in African nations has developed herd immunity to COVID-19 due to their earlier exposure to the Ebola virus (32, 33). Finally (34, 35), corroborated that certain live attenuated vaccines (BCG vaccine, oral polio vaccine, and measles vaccines) elicit strong non-specific innate immune responses that also confer protection against COVID-19 in the African population. Despite the low number of deaths in Africa, the impact of COVID-19 deaths has been profound. According to (2, 36), the death toll in several African countries incurred economic costs due to the predominance of trained and capable individuals among the victims. The situation created a skills deficit for the economy and resulted in futile medical treatment expenses for the victims’ survival.

Given this circumstance, time series intervention analysis of COVID-19 deaths appears to provide valuable insights for governments and the global community in forecasting and controlling COVID-19 fatalities. By evaluating the impact of specific safety protocols, such as social distancing and behavior change campaigns, governments can formulate effective strategies for pandemic management (37), demonstrated this through segmented regression analysis in South Korea, showing an apparent decrease in COVID-19 cases during periods of strict social distancing, followed by an increase in policy relaxation. This study, along with findings from (38, 39), corroborates the effectiveness of social distancing in reducing infectious disease transmission during outbreaks, highlighting its crucial role in mitigating hospital admissions and controlling the spread of the pandemic. The focus of time series intervention analysis of COVID-19 deaths has been given little attention in the literature.

Despite a growing body of literature on COVID-19 modelling, most of the studies (40–44) predominantly concentrated on disease transmission, mitigation strategies, social, and economic impact rather than the quantification and distribution of deaths experienced during the crisis period. Various statistical and mathematical techniques were employed to conduct a range of time series intervention analyses. For instance (40), developed a SEIQHRS (susceptible-exposed-infectious-quarantine-hospitalized-recovered-susceptible) model that predicts the trajectory of the COVID-19 pandemic, aiding in the formulation of an effective control strategy for COVID-19 in Ghana. Their findings suggested that COVID-19 deaths decreased with the introduction of safety protocols. However, the mitigation measures studied independently could not support a reduction in the reproductive number (44), employed a generalized linear time-series regression model to assess the effectiveness of the government’s decision to reopen the country’s borders during the spread of COVID-19. The study concluded that the mitigation strategies were somewhat effective. Conversely (41), observed an erratic pattern in the transmission of the disease as well as death cases, irrespective of the interventions implemented. Therefore, understanding and predicting monthly mortality trends is therefore critical for resource allocation, especially during public health crises such as the COVID-19 pandemic.

Most Ghanaian COVID-19 modelling studies reviewed focus on case counts or short-term epidemic projections, often omitting the indirect mortality effects and the broader all-cause perspective. In this paper, we aim to examine the impact of the COVID-19 pandemic, applying time series intervention analysis to provide evidence of a change in trend in the death count for Ghana. Consequently, we wish to test the hypothesis that the COVID-19 pandemic significantly increased the total number of deaths in Ghana. According to (45), intervention refers to an event, procedure, or process that alters the values of a time series. We further illustrate the incidence of the COVID-19 pandemic as an intervention by a time series analysis, employing the total death count data from Ghana. The novelty of our approach lies in combining COVID-19 and non-COVID-19 deaths into a single all-cause mortality forecast, enabling a comprehensive assessment of the pandemic’s total mortality impact in Ghana. By leveraging routinely collected civil registration data, this study demonstrates a pragmatic, locally relevant application of time-series forecasting to inform national public health planning.

## 2 Methods

The study employed the Interrupted Time Series (ITS) design, a quasi-experimental approach, using monthly all-cause mortality data to develop and validate an ARIMA model. This design is ideal for evaluating the impact of a naturally occurring event, or “intervention,” on a trend over time (45). The analysis covered the period from January 2018 to December 2022, encompassing both pre-pandemic and pandemic periods. The monthly total death time series data for the study were obtained from the Ghana’s Birth and Death Registry (BDR). The BDR is the official governmental body responsible for compiling birth and death statistics in Ghana. The Registry compiles registrations from across all regions. The Registry operates under the Act 1027 of 2020, within the Ministry of Local Government and Rural Development of Ghana and adopt the District Health Information Management System (DHIMS2) to support public health reporting. Monthly death counts were extracted from aggregated administrative records and anonymised prior to analysis. Data underwent standard completeness and consistency checks as part of routine registry procedures.

Modelling all-cause mortality without distinguishing between COVID-19 and non-COVID-19 fatalities is justified by various valid methodological and practical considerations. This data are generally more timely and reliable than cause-specific data, which often encounter delays or misclassification during crisis periods, like as the COVID-19 pandemic (46). The aggregated data types have been extensively used for real-time forecasting and excess mortality estimation in several scenarios, including during COVID-19 surges; these methodologies are well established in epidemiological modelling (47). Furthermore, excess mortality frameworks, supported by organisations such as the WHO, acknowledge that integrating both direct and indirect pandemic-related deaths with other mortality statistics is essential for capturing the pandemic’s full impact, which cause-specific data may underrepresent (48). Collecting all-cause data without differentiating the cause of death is a reasonable and conventional approach for projecting population-level mortality, particularly when timely and comprehensive insights are necessary.

The emergence of COVID-19 pandemic variants, such as Delta and Omicron, with differing transmissibility and mortality profiles, is recognized as having significantly influenced the epidemic dynamics. However, the study’s objective was to model aggregate mortality statistics rather than variant-specific epidemiological behavior. According to (47), when reliable, variant-stratified mortality data for the entire forecasting period are unavailable across reporting systems, making direct inclusion of variant variables infeasible without introducing significant bias. In the case of this study, the effect of emerging variants is indirectly captured in the historical mortality data used for model training, which inherently captures the combined impact of transmission dynamics, healthcare system responses, and changes in mortality risk over time. This approach, while not variant-specific, has been widely applied in mortality forecasting during evolving pandemic phases (46).

As a result, the data for the study covers the period from January 2018 to December 2022. Data from January 2018 to March 2020 was utilized to construct the pre-intervention time series model. Consequently, the forecast made beyond March 2020 constitutes an out-of-sample prediction. In this context, the pre-intervention period refers to all data points before March 2020, representing baseline mortality trends unaffected by the COVID-19 pandemic, while the post-intervention period covers data from March 2020 onward, reflecting the pandemic’s potential impact. The pre-intervention data are used to estimate the model parameters, and the fitted model is then applied to predict mortality in the post-intervention period, enabling assessment of any deviations attributable to the pandemic. Literature (45, 49, 50) provides that a reasonable ARIMA model requires at least 40 to 50 observations. This study satisfies this condition as we used 60 data points for our analysis. Table 1 presents the total monthly deaths in Ghana from January 2018 to December 2022 as obtained from Ghana’s BDR.

**Table 1:** Monthly deaths in Ghana from January 2018 to December 2022.

	Monthly deaths											
Year	Jan	Feb	Mar	Apr	May	Jun	Jul	Aug	Sep	Oct	Nov	Dec
2022	4,721	4,330	4,810	3,778	3,667	3,733	4,218	4,442	4,263	4,673	4,664	3,648
2021	4,437	4,938	5,095	4,636	4,160	4,174	4,619	4,958	5,047	4,676	4,694	3,915
2020	4,805	4,447	3,544	3,742	4,206	4,205	4,441	4,458	4,520	4,319	4,591	3,748
2019	4,737	4,152	4,192	3,950	4,327	4,018	4,532	4,107	4,149	4,326	4,211	4,353
2018	4,863	4,120	3,837	4,223	4,098	3,819	3,981	4,377	4,182	4,363	4,393	3,373

The casual visualization of the data failed to adequately convey the impact of COVID-19 on the death count in Ghana. Therefore, we proceeded to find statistical techniques that can provide a suitable evaluation of the pandemic’s effect on the death count in Ghana. One such technique is the autoregressive integrated moving averages (ARIMA) (51), have documented the usefulness of ARIMA modelling in assessing health interventions. According to (52), the ARIMA model is advantageous for time series data that demonstrates a non-linear trend, probable seasonality, or periodicity, rendering it highly adaptable. In assessing the performance of the ARIMA model (53), deployed autoregressive (AR), moving average (MA), autoregressive moving average (ARMA), and ARIMA models to forecast the spread of COVID-19 in Saudi Arabia. The performance of each model was assessed using the RMSE, MAE, coefficient of determination ( $R^{2}$ ), MAPE, and root mean squared relative error (RMSRE) performance metrics. Alzahrani et al. assert that the ARIMA model outperforms other models by achieving the lowest RMSE, MAPE, and RMSRE values, along with the highest $R^{2}$ value (53). Time series intervention analysis has been employed to address various problems involving time series data (45, 54–58). The COVID-19 pandemic has also been studied from various perspectives (59, 60). However, none of these articles applies ARIMA with intervention analysis to assess the impact of the COVID-19 pandemic on the number of deaths.

This study examines a specific scenario where the input consists of an indicator variable representing the occurrence and impact of the COVID-19 pandemic on the number of deaths in Ghana. The COVID-19 pandemic serves as the identified single intervention event occurring within the time under study. We posit that an intervention has occurred; therefore, we examine whether evidence exists indicating that the anticipated change in the time series has taken place. Additionally, we enquire about the projected magnitude of this alteration. We examine the three phases of ARIMA modelling to identify the order of differencing. This initial phase employs the Kwiatkowski–Phillips–Schmidt–Shin (KPSS) test to generate a stationary time series model for identification purposes. All statistical computations, probability estimations, and graphical analyses were performed using the RStudio statistical software version 2023.12.0.

Secondly, we provisionally diagnose and identify the parameters of the autoregressive and moving-average components of the ARIMA model by applying the autocorrelation function (ACF). Thirdly, we assess the residuals to evaluate the model’s fit to the data using the ACF of the residuals.

### 2.1 Time series intervention analysis

Suppose that at time *t* = *T* (where *T* is known), there has been an intervention in a time series. By intervention, we refer to a modification of a procedure, law, policy, or other measures aimed at altering the values of the series $y_{t}.$ Intervention analysis in time series involves examining alterations in the mean level of a series following an intervention, assuming that the same ARIMA structure applies both before and after the intervention. The ARIMA (*p*, *d*, *q*) model, with no intervention, is written as


(1) $$y_{t}^{\prime }=\phi_{1}y_{t-1}^{\prime }+\ldots +\phi_{p}y_{t-p}^{\prime }+\theta_{1}\varepsilon_{t-1}+\ldots +\theta_{q}\varepsilon_{t-q}+\varepsilon_{t},$$

where $y_{t}^{\prime }=y_{t}-y_{t-1}$ is the differenced series (it may have been differenced more than once), the error term $\varepsilon_{t}\overset{\mathrm{iid}}{∼}N(0,\sigma_{\varepsilon }^{2})$ and *p* = order of the autoregressive part, *d* = degree of first differencing involved, *q* = order of the moving average part. Equation 1 can be written as


$$\Phi \;(B)\;{\left(1-B\right)}^{d}y_{t}=\Theta \;(B)\;\varepsilon_{t},$$

$$y_{t}={\left(1-B\right)}^{-d}\Phi^{-1}(B)\;\Theta \;(B)\;\varepsilon_{t},$$

where *B* is the backshift operator, Φ(B), and Θ(B) are the AR and MA polynomials, respectively. These functions are defined by $\Phi (B)=(1-\phi_{1}B-\phi_{2}B^{2}-\cdots -\phi_{q}B^{q})$ and $\Theta (B)=(1+\theta_{1}B+\theta_{2}B^{2}+\cdots +\theta_{q}B^{q}).$ Let $a_{t}$ be the amount of change at time *t* that is attributable to the intervention. By definition, $a_{t}$ is zero before time *T* (time of the intervention). The value $a_{t}$ may or may not be 0 after time *T*. According to (45), the overall model, including the intervention effect, is written as:


$$z_{t}=a_{t}+y_{t}.$$

**Figure 1:**
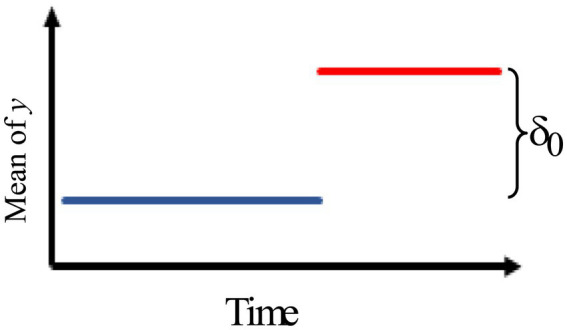
Intervention effect model with a permanent constant change in level of magnitude.

There are several different response patterns, $a_{t},$ for how an intervention may affect the values of a series for *t* ≥ *T* (the intervention point). Four possible patterns are as follows:

1.Figure 1 describes an intervention effect model with a permanent constant change in level of magnitude of $\delta_{0}$ after time 𝑇.

We can describe the impact of the intervention model as


$$a_{t}=\{\begin{array}{l} 0,\;\;\;\;t<T\\ \delta_{0},\;\;t\geq T. \end{array}$$

**Figure 2:**
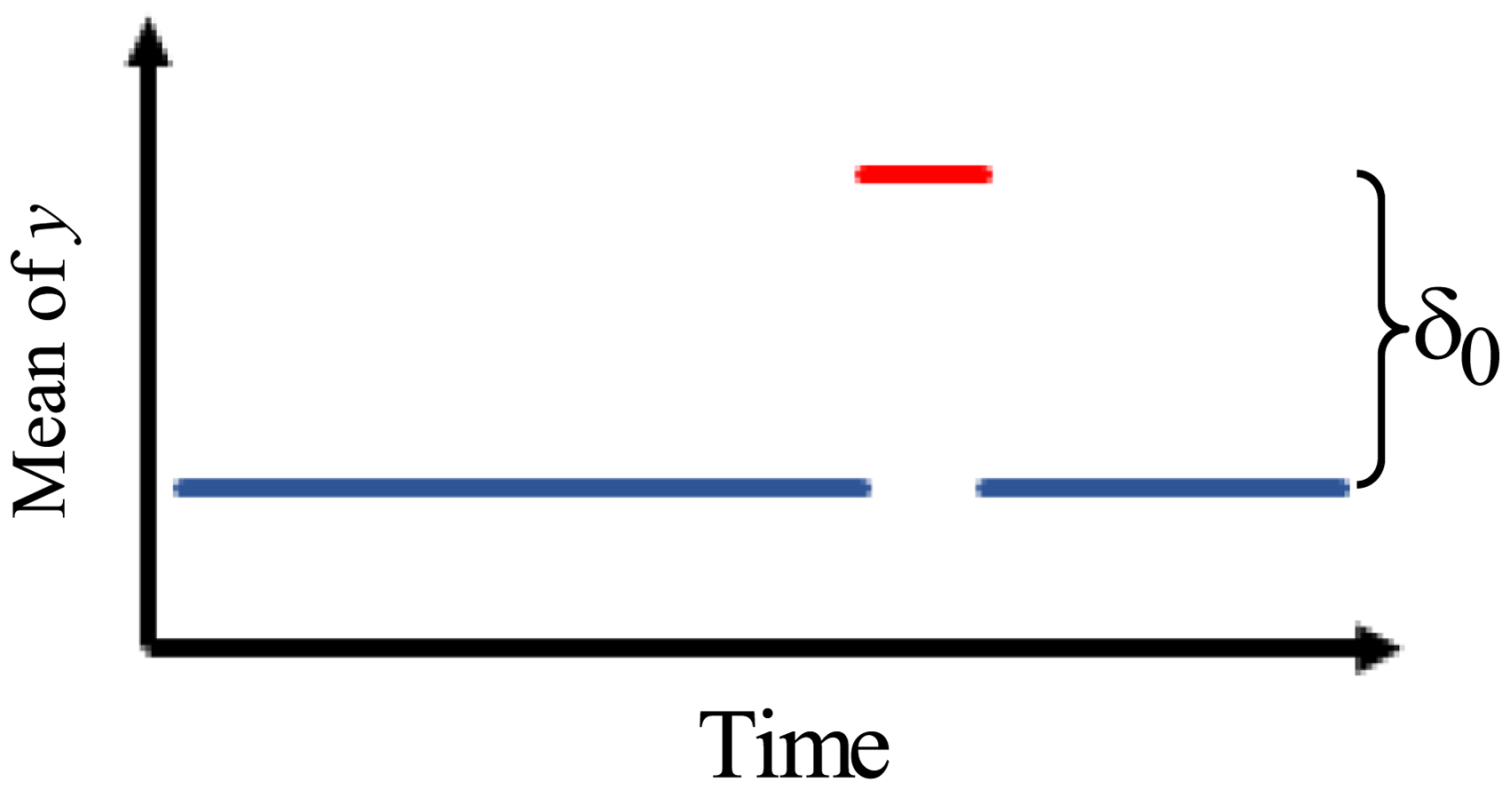
Intervention effect model with a brief constant change.

2.Figure 2 describes an intervention effect model with a brief constant change lasting for δ*t* times past the intervention time *T*.

Thus, the intervention effect model is given by


$$a_{t}=\{\begin{array}{l} \delta_{0},T<t\leq T+\mathrm{\delta t}\\ 0,\;\;\;\text{elsewhere}. \end{array}$$

**Figure 3:**
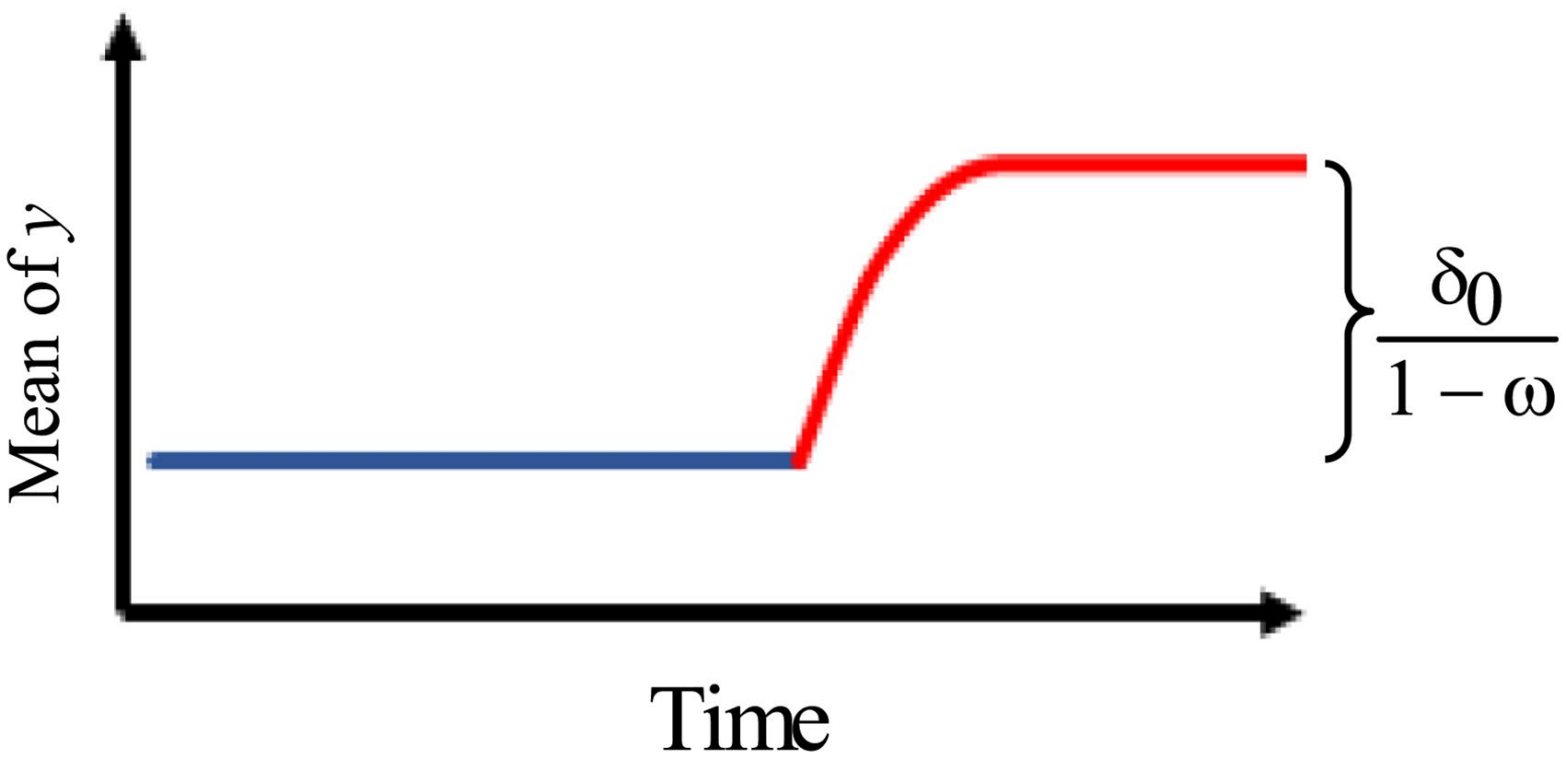
Intervention effect model with a gradually increasing effect of the rate *ω*.

3.Figure 3 describes an intervention model with a gradually increasing effect of the rate *ω* that eventually levels off.

Thus, we use the model:


$$a_{t}=\{\begin{array}{l} 0,\;\;\;t<T\\ \frac{\delta_{0}(1-\omega^{t-T})}{1-\omega },\;\;t\geq T. \end{array}$$

where ∣ω∣<1.

**Figure 4:**
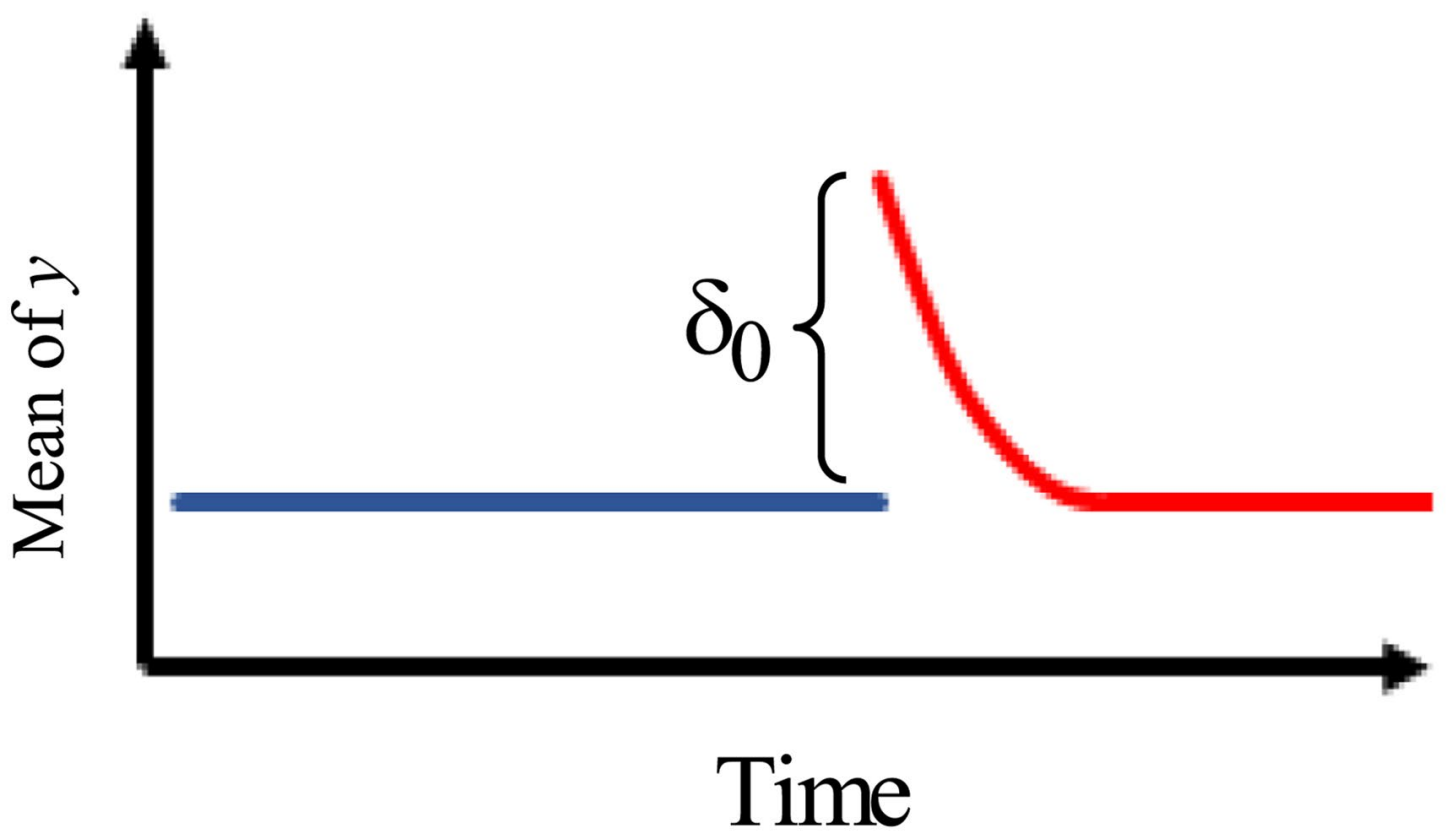
Intervention effect model with an immediate change, followed by a gradual decay of rate back to the original pre-intervention level.

4.Figure 4 describes an intervention model with an immediate change, followed by a gradual decay of rate ω back to the original pre-intervention level with no permanent effect.

The model is, therefore, given by


$$a_{t}=\{\begin{array}{l} 0,\;\;\;\;t\leq T\\ \delta_{0}\omega^{t-T-1},t>T \end{array}$$

where ∣ω∣<1.

### 2.2 Estimating the intervention effect

We consider the simple model


(2) $$z_{t}=\delta_{0}I_{t}+y_{t},$$

where $I_{t}$ is an indicator variable such that $I_{t}$ = 1 when *t* ≥ *T* and $I_{t}$ = 0 when *t* < *T*, and $y_{t}={\left(1-B\right)}^{-d}\Phi^{-1}(B)\;\Theta \;(B)\;\varepsilon_{t}.$ This model can be written, formally, as


(3) $$\pi (B)z_{t}=\delta_{0}\;\pi (B)\;I_{t}+\varepsilon_{t},$$

where $\pi (B)={\left(1-B\right)}^{d}\Phi (B)\;\Theta^{-1}(B)=1-\sum_{i=1}^{\infty }\pi_{i}B^{i}.$ Letting $u_{t}=\pi (B)I_{t}$ and $v_{i}=\pi (B)y_{t},$ we can write Equation 3 in the form of a simple linear model $v_{i}=\delta_{0}u_{t}+\varepsilon_{t},t=1,2,\ldots ,n$ . Hence, the maximum likelihood estimator of $\delta_{0}$ is approximately ${\hat{\delta }}_{0}=\sum_{i=1}^{n}u_{t}v_{t}/\sum_{i=1}^{n}u_{t}^{2}$ with $\mathrm{var}({\hat{\delta }}_{0})=\sigma_{\varepsilon }^{2}/\sum_{i=1}^{n}u_{t}^{2}.$

## 3 Results and discussion

Initially, we used the information presented in Table 1, designating March 2020 as the single intervention point (the month marking the commencement of the COVID-19 pandemic in Ghana), to determine the ARIMA model for the pre-pandemic series.

The Kwiatkowski Phillips Schmidt Shin (KPSS) test, conducted to assess the stationarity of the pre-intervention data with a lag order of two, yielded a *p*-value of 0.063, indicating, at the 5% level of significance, that the series is stationary. A superior result was obtained after performing a first-order difference of the data. The KPSS test produced a p-value of less than 0.077 with a lag of order two. This result shows that the first-order difference of the pre-pandemic data is stationary. Since we had to ‘difference’ the data once, the *d* value for our ARIMA (*p*, *d*, *q*) model resulted in one.

Table 2 presents a comparative analysis of the ARIMA models: ARIMA (1, 1, 0), ARIMA (2, 1, 0), and ARIMA (3, 1, 0). The Akaike Information Criterion (AIC) and Bayesian Information Criterion (BIC) for ARIMA (3, 1, 0) are lower than those for ARIMA (2, 1, 0) and ARIMA (1, 1, 0). Therefore, the optimal model is ARIMA (3, 1, 0).

**Table 2:** Comparative analysis of ARIMA (2, 1, 0), ARIMA (2, 1, 0) and ARIMA (3, 1, 0).

Model Components	ARIMA (1, 1, 0)			ARIMA (2, 1, 0)			ARIMA (3, 1, 0)		
	Estimate	SE	*p*-value	Estimate	SE	*p*-value	Estimate	SE	*p*-value
AR(1)	−0.5594	0.1756	0.0041	−0.8818	0.2089	0.0004	−1.1013	0.2197	0.0001
AR(2)				−0.4893	0.2044	0.0256	−0.9100	0.2683	0.0027
AR(3)							−0.4355	0.2041	0.0448
Constant	−6.1980	52.7626	0.9075	4.4404	31.8013	0.8902	9.4769	20.3814	0.6467
AIC	15.09849			14.98564			14.91084		
BIC	15.24475			15.18066			15.15462		

**Figure 5:**
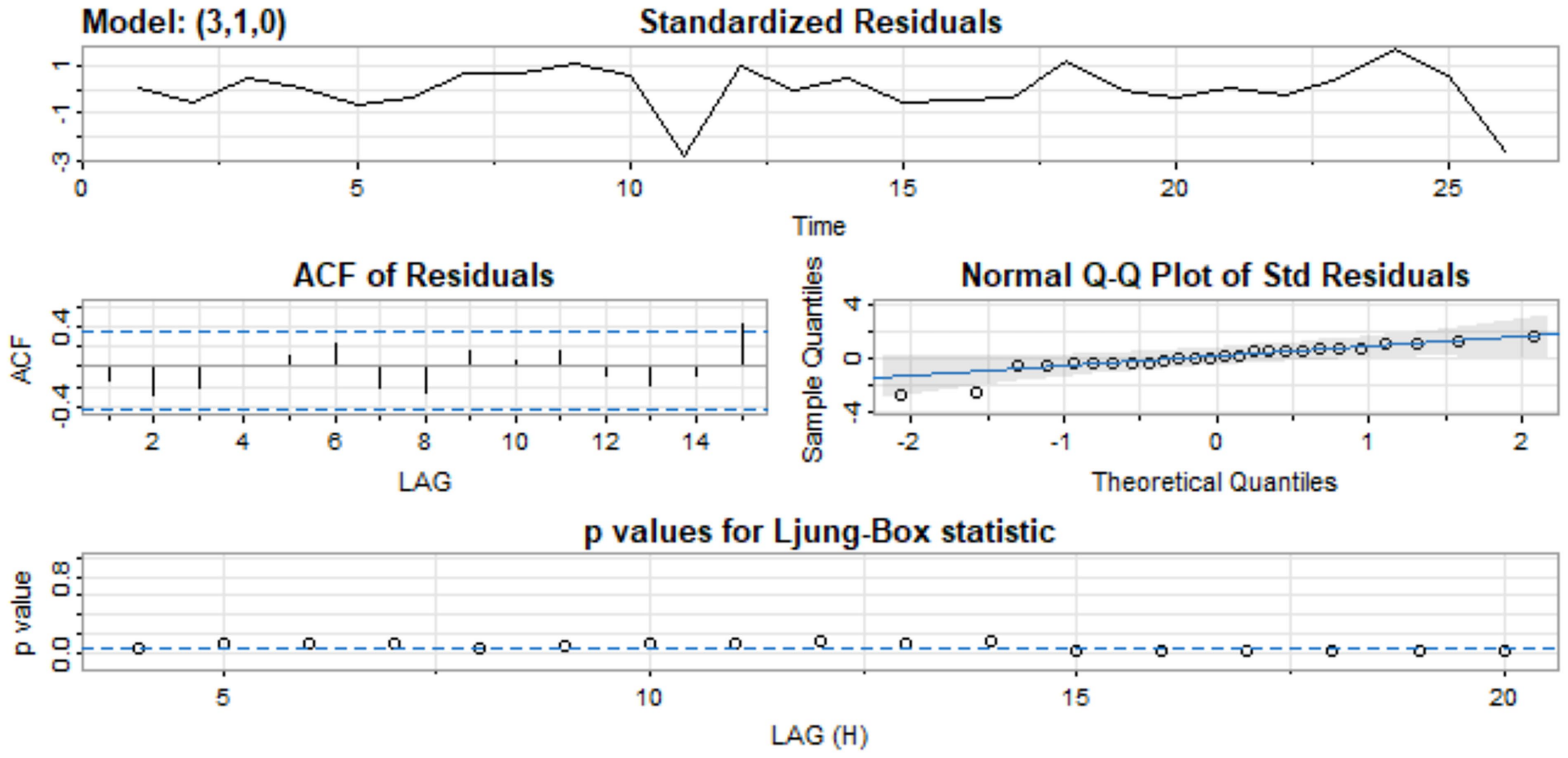
Standardized residual, ACF of residual, normal Q-Q of standard residuals, and *p*-values of Ljung-Box statistic.

Figure 5 shows the standardized residual plot for the ARIMA (3, 1, 0) model, accompanied by the Q-Q normality plot, the ACF residual plot, and the p-value plot of the Ljung-Box statistic. The standardized residuals plot demonstrates the absence of a trend in the residuals and, in general, no change in variance with time. The ACF of the residuals shows no significant autocorrelations. In the Q-Q plot, the points appear to align along a straight trajectory. As a result, the residuals exhibit a normal distribution. The *p*-value for the Ljung-Box statistic at lag 20 is 0.5228, indicating that the residuals are independently distributed. Therefore, we conclude that the model does not exhibit a lack of fit.

We employ the ARIMA (3, 1, 0) model to forecast values for the period after the intervention point and subsequently compute the differences between the observed and the predicted values. The observed values represent the actual total deaths recorded after the intervention point. The predicted values are the estimates generated by our ARIMA (3, 1, 0) model. The predicted values are expected to be lower than the observed values if, and only if, the COVID-19 pandemic indeed caused a higher number of deaths, as observed in regions such as Europe, America, and Asia (1).

Table 3 presents the observed and predicted values for the period following the intervention point along with the observed absolute percentage differences.

**Table 3:** The predicted and observed values after the intervention point.

Month-year	Observed	Predicted	Difference	% Difference	Month-year	Observed	Predicted	Difference	% Difference
May-20	4206.0	4437.3	−231.3	5.5	Sep-21	5047.0	4452.7	594.3	11.8
Jun-20	4205.0	4100.5	104.5	2.5	Oct-21	4676.0	4465.3	210.7	4.5
Jul-20	4441.0	4239.7	201.3	4.5	Nov-21	4694.0	4464.4	229.6	4.9
Aug-20	4458.0	4540.0	−82.0	1.8	Dec-21	3915.0	4476.5	−561.5	14.3
Sep-20	4520.0	4262.0	258.0	5.7	Jan-22	4721.0	4491.2	229.8	4.9
Oct-20	4319.0	4266.9	52.1	1.2	Feb-22	4330.0	4497.1	−167.1	3.9
Nov-20	4591.0	4416.4	174.6	3.8	Mar-22	4810.0	4504.6	305.4	6.3
Dec-20	3748.0	4401.0	−653.0	17.4	Apr-22	3778.0	4517.2	−739.2	19.6
Jan-21	4437.0	4312.4	124.6	2.8	May-22	3667.0	4526.6	−859.6	23.4
Feb-21	4938.0	4391.5	546.5	11.1	Jun-22	3733.0	4534.2	−801.2	21.5
Mar-21	5095.0	4424.4	670.6	13.2	Jul-22	4218.0	4544.5	−326.5	7.7
Apr-21	4636.0	4387.5	248.5	5.4	Aug-22	4442.0	4554.8	−112.8	2.5
May-21	4160.0	4396.4	−236.4	5.7	Sep-22	4263.0	4563.4	−300.4	7.0
Jun-21	4174.0	4438.5	−264.5	6.3	Oct-22	4673.0	4572.7	100.3	2.1
Jul-21	4619.0	4432.8	186.2	4.0	Nov-22	4664.0	4582.8	81.2	1.7
Aug-21	4958.0	4429.6	528.4	10.7	Dec-22	3648.0	4592.1	−944.1	25.9
					**Total**	**140784.0**	**142217.0**	**−1433.0**	**263.8**
					**Mean**	**4399.5**	**4444.3**	**−44.8**	**8.2**

It can be deduced from Table 3 that 22 of the 32 estimated values fall within 10% of the actual values for the post-intervention period, with 15 within 5% and 7 exhibiting errors between 10 and 20%. The sum of the death counts during the post-intervention period gave a value of 140,784 (the observed). When we used our estimated pre-intervention ARIMA model to calculate the total number of post-intervention deaths, the result was 142,217. The difference (−1433.0) indicates fewer deaths in the post-intervention period, consistent with (40), which suggests that COVID-19 deaths decreased following the introduction of safety protocols. This finding contrasts with the anticipated increase in deaths resulting from the COVID-19 pandemic. Similarly, during the post-intervention period, the average observed death count was 4399.5 compared to the estimated average predicted deaths of 4444.3. The difference between these two averages provides an assessment of the causal impact of the COVID-19 pandemic on the number of deaths in Ghana.

The effect is −44.8, representing a decrease of approximately 1% in the number of deaths. From Equation 2, it can be inferred that $\delta_{0}$ measures the intervention effect. Its estimate determines the magnitude of change between the average observed deaths and the average predicted death counts. Consequently, the estimated intervention effect, ${\hat{\delta }}_{0},$ is −44.8. Thus, representing a marginal difference of about 1% in death counts. This decrease in total death count means a marginal number of lives were saved during the period understudy. This translates to a substantial number of lives, raising a public health intervention issue. Thus, freeing some level of burden on the healthcare system in Ghana. The implication is that if the population of Ghana is approximately 35 million people, with an average annual death rate of approximately 7 deaths per 1,000 people. Then, it can be estimated that Ghana had averagely, 245,000 deaths annually. Therefore, a decrease of 1% in the number of deaths would mean 2,450 lives were saved.

Furthermore, we tested the significance of this marginal difference between the expected and the observed death counts. We used both the paired t-test and the Wilcoxon matched-pairs signed-rank test. Prior to the paired t-test, we obtained a Shapiro–Wilk normality test statistic of 0.945 and a corresponding *p*-value of 0.101. This result also shows that the differences found in Table 3 conform to a normal distribution. Hence, the paired sample *t*-test produced a test statistic of −0.584 with a p-value of 0.563. The findings indicate that there is no significant difference between the observed and the predicted death toll for the post-intervention period. Alternatively, the Wilcoxon matched-pairs signed-rank test also confirmed this conclusion with a test statistic of 283 and a p-value of 0.722.

**Figure 6:**
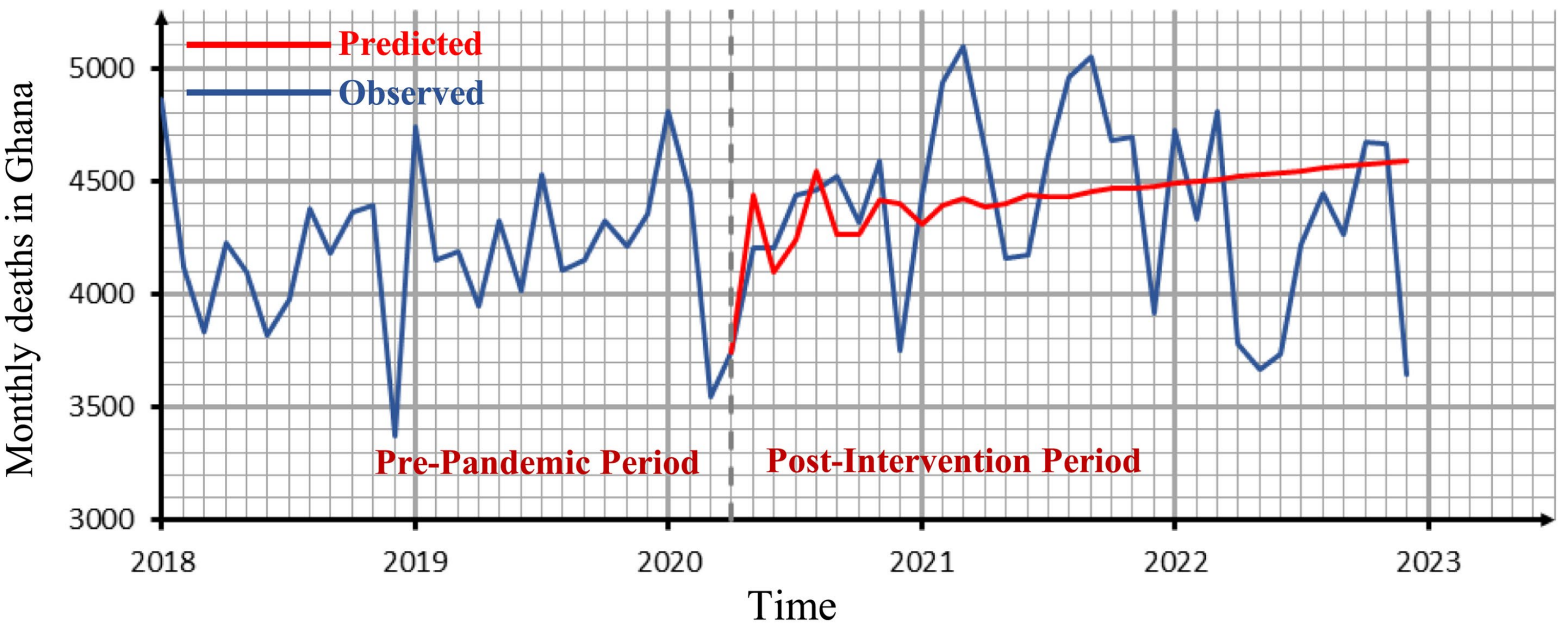
The observed time series graph with the forecasted values for the period after the intervention point.

Figure 6 shows our original data set and predicted values for the period after the intervention point based on the pre-pandemic ARIMA (3, 1, 0) model. The ARIMA model’s effectiveness in this study is consistent with prior applications in health-related time-series forecasting. Similar success has been reported in China (10), across nine countries in Europe, Asia, and the Americas (11), and in Nigeria (14), underscoring its suitability for short-term mortality prediction even in resource-limited settings.

This study examines the trends in road traffic fatalities (RTF) in Ghana from 1990 to 2024 in order to determine whether the COVID-19 lockdowns may have impacted overall mortality trends.

**Figure 7:**
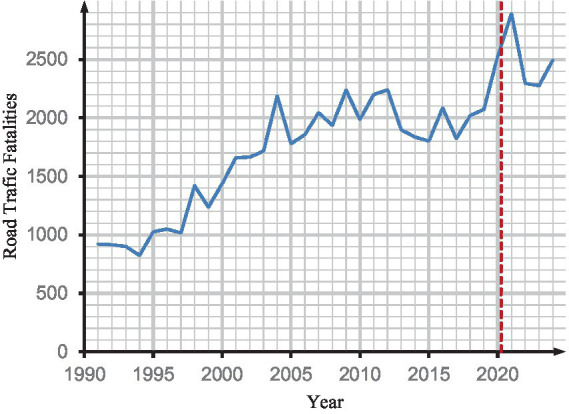
Changes in national road traffic fatality in Ghana.

The purpose of this is to eliminate the possibility of a confounding decrease in mortality that is attributable to other factors, such as lockdowns. It was crucial to evaluate whether any decrease in RTF could have counteracted increases in COVID-19-related mortality, potentially concealing excess deaths during the pandemic, as lockdowns were anticipated to reduce traffic accidents and, as a result, fatalities from RTF. Nonetheless, as shown in Figure 7, RTF continued to rise throughout the pandemic period, indicating that the lack of observed excess mortality in our results is unlikely to be explained by reductions in traffic accident deaths.

While Ghana’s Birth and Death Registry applies WHO standards and uses DHIMS2 to support public health reporting, this study acknowledges potential challenges including the reliability of death registration data, possible cause-of-death misclassification or underreporting, and the influence of seasonal or regional reporting variations. To address these issues, sensitivity analysis was performed to evaluate the robustness of our findings. Specifically, post-intervention mortality figures were adjusted by ±10% to account for potntial underreporting or overreporting, allowing us to assess the impact of missing or misclassified data on the results. In this analysis, independent sample t-tests were used to compare pre-intervention and post-intervention residuals across two scenarios: a 10% downward adjustment and a 10% upward adjustment. The pre and post intervention residuals in the −10% adjusted dataset had different means, but there was no significant difference between the residuals [*t* = 1.13, *p* = 0.2633, 95% CI: (−74.39, 266.95)]. Likewise, no significant difference was observed in the +10% scenario [*t* = −0.14, *p* = 0.8882, 95% CI: (−216.10, 187.64)]. Across both scenarios, the t-test consistently indicated no significant differences between the pre- and post-intervention residuals. This suggests that:

- The intervention had no detectable impact on residual patterns.
- The analysis is robust to ±10% modifications in post-intervention data, accounting for possible underreporting or overreporting.

To determine if the available sample size was sufficient to detect a significant difference between groups (pre- and post-intervention), a power analysis was conducted. According to the findings, the study’s statistical power to identify a modest effect size (Cohen’s *d* = 0.2) was 85.8%. This degree of power implies that the sample sizes (*n*_1_ = 27 and *n*_2_ = 33) were sufficient to detect any differences, regardless of their magnitude. The dependability of the test results was further supported by the comparatively low Type II error rate (the likelihood of missing a true effect), which was 14.2%. These findings demonstrate that the analysis was well-powered to identify subtle effects at the selected significance level, which is crucial for research where even modest variations could have theoretical or practical implications. As a result, a statistically significant change is likely to be indicative of a major impact. Conversely, if no significant difference is observed, it strengthens the interpretation that any potential differences between the groups are likely negligible rather than missed due to insufficient sample size.

The outcome of the study verifies that the COVID-19 pandemic did not significantly influence the monthly death toll in Ghana. This result is consistent with previous studies such as (40, 41, 44), which indicated no definitive evidence that the COVID-19 pandemic led to an increase in number of deaths in Ghana during the period under examination. The minimal change noted in this study, which diverges from global expectations of elevated excess mortality, may be attributed to public health measures, demographic robustness, a reduced incidence of severe COVID-19 comorbidities, or the possibility that Africa’s youthful demographic is acquiring herd immunity from previous infections. These factors highlight the need for pandemic preparedness strategies tailored to the sub-Saharan African context.

## 4 Conclusion and recommendation

The rapid spread of the COVID-19 pandemic resulted in numerous fatalities worldwide from the time of the commencement of the epidemic to the end of December 2021. The primary objective of our study is to examine the impact of the pandemic on the number of deaths in Ghana using an ARIMA model with intervention analysis applied to monthly death data from January 2018 to December 2022. The ARIMA modeling framework was selected for its reliability and widespread use in analyzing time series data involving discrete interventions. March 2020, when COVID-19 was officially recorded in Ghana, provided a distinct temporal reference point. This made it suitable to model the pandemic’s impact as a discrete shock within the ARIMA framework. ARIMA’s adaptability in detecting patterns, capturing autocorrelation, and accounting for both short-term shocks and long-term trends further justified its application to the mortality data. We recognize that the pandemic’s impact on mortality is unlikely to manifest as a single abrupt change. The effects could have evolved gradually, influenced by multiple overlapping waves, behavioral shifts, and public health policies. Future studies may benefit from applying models better suited to capturing gradual or multiple interventions, such as segmented regression or Bayesian structural time series approaches.

Selecting ARIMA parameters (p, d, q) presents several challenges, especially for noisy or limited datasets. This may lead to overfitting or underfitting, therefore affecting model interpretability and accuracy. To address this, our model selection was supported by diagnostic tests, including ACF plots, the Ljung–Box test, KPSS test, and residual analysis, which assessed stationarity and ensured residuals approximated white noise. The selection of the model was additionally reinforced by employing the AIC and the BIC to determine the most parsimonious and well-fitting model.

Additionally, both parametric (paired t-test) and non-parametric (Wilcoxon signed-rank test) methods were applied to compare forecasted and observed deaths post-intervention. Mortality during the study period aligned closely with patterns predicted from historical data. The absence of substantial change identified in this study may be attributed to the implemented safety protocols or the belief that the younger demographic in African countries is acquiring herd immunity to COVID-19 due to prior exposure to other infections. While our findings may be reassuring, maintaining vigilance remains crucial to prevent future threats and strengthen data systems for more resilient decision-making. Continued vigilance and sustained investment in data systems and public health infrastructure are essential, along with ongoing surveillance, excess mortality monitoring, and capacity-building for analytical modeling to effectively detect and respond to future public health shocks.

## Data Availability

The original contributions presented in the study are included in the article/supplementary material, further inquiries can be directed to the corresponding author/s.
